# Origin of Brandy

**Published:** 1865-02

**Authors:** 


### ORIGIN OF BRANDY.

Brandy began to be distilled in France about the year 1313, but it was prepared only as a medicine, and was considered as possessing such marvellous strengthening and sanitary powers that the physicians named it "the water of life," (l'eau de vie,) a name it still retains, though now rendered, by excessive potations, one of life's most powerful and prevalent destroyers. Raymond Lully, a disciple of Arnold de Villa Nova, considers this admirable essence of wine to be an emanation from the Divinity, and that it was intended to re-animate and prolong the life of man. He even thought that this discovery indicated that the time had arrived for the consummation of all things—the end of the world. Before the means of determining the true quantity of alcohol in spirits were known, the dealers were in the habit of employing a very rude method of forming a notion of the strength. A given quantity of the spirits was poured upon a quantity of gunpowder in a dish and set on fire. If at the end of the combustion the gunpowder continued dry enough, it exploded, but if it had been wetted by the water in the spirits, the flame of the alcohol went out without setting the powder on fire. This was called the proof. Spirits which kindled gunpowder were said to be above proof.

From the origin of the term "proof," it is obvious that its meaning must at first have been very indefinite. It could serve only to point out those spirits which are too weak to kindle gunpowder, but could not give any information respecting the relative strength of those spirits which were above proof. Even the strength of proof was not fixed, because it was influenced by the quantity of spirits employed—a small quantity of weaker spirit might be made to kindle gunpowder, while a greater quantity of a stronger might fail. Clark in his hydrometer, which was invented about the year 1730, fixed the strength of proof-spirits on the stem at the specific gravity of 0.920 at the temperature of 60 degrees. This is the strength at which proof-spirit is fixed in Great Britain by Act of Parliament, and at this strength it is no more than a mixture of 49 pounds of pure alcohol with 51 pounds of water. Brandy, rum, gin, and whiskey contain nearly similar proportions.

